# ASM Based Synthesis of Handwritten Arabic Text Pages

**DOI:** 10.1155/2015/323575

**Published:** 2015-07-30

**Authors:** Laslo Dinges, Ayoub Al-Hamadi, Moftah Elzobi, Sherif El-etriby, Ahmed Ghoneim

**Affiliations:** ^1^Institute for Information Technology and Communications (IIKT), Otto-von-Guericke-University Magdeburg, 39016 Magdeburg, Germany; ^2^Umm Al-Qura University, Makkah 21421, Saudi Arabia; ^3^Faculty of Computers and Information, Menoufia University MUFIC, Menofia 32721, Egypt; ^4^Department of Software Engineering, College of Computer Science and Information Sciences, King Saud University, Riyadh 11451, Saudi Arabia; ^5^Department of Computer Science, College of Science, Menoufia University, Menofia 32721, Egypt

## Abstract

Document analysis tasks, as text recognition, word spotting, or segmentation, are highly dependent on comprehensive and suitable databases for training and validation. However their generation is expensive in sense of labor and time. As a matter of fact, there is a lack of such databases, which complicates research and development. This is especially true for the case of Arabic handwriting recognition, that involves different preprocessing, segmentation, and recognition methods, which have individual demands on samples and ground truth. To bypass this problem, we present an efficient system that automatically turns Arabic Unicode text into synthetic images of handwritten documents and detailed ground truth. Active Shape Models (ASMs) based on 28046 online samples were used for character synthesis and statistical properties were extracted from the IESK-arDB database to simulate baselines and word slant or skew. In the synthesis step ASM based representations are composed to words and text pages, smoothed by B-Spline interpolation and rendered considering writing speed and pen characteristics. Finally, we use the synthetic data to validate a segmentation method. An experimental comparison with the IESK-arDB database encourages to train and test document analysis related methods on synthetic samples, whenever no sufficient natural ground truthed data is available.

## 1. Introduction

A crucial step for every pattern recognition system is to train the classifier against a database and validate the system using the corresponding ground truth (GT). However, collecting handwriting samples is known as error prone, labor-, and time-expensive process [1]. Particularly costly are databases, which are suitable for training and validation of methods, that segment words into letters or analyse text pages as historical documents, since corresponding GT needs to include additional information. For real handwriting databases this has to be done in a time consuming manual or semimanual way. This is one of the reasons, why data synthesis recently gained more and more interest [2].

The problem of the lack of satisfactory handwriting databases is very obvious in case of Arabic handwritings, where there mainly two well known, free available (offline) word databases (IFN/ENIT) [3], which exclusively contains Tunisian town names, and the IESK-arDB [4] that contains international town names and common terms as well, as 280 historical manuscript pages and 6000 segmented characters. We developed the IESK-arDB word database as a general database to train and validate segmentation based recognition of Arabic words. Therefore, the writers are from different Arabic countries; however, all of them writing in standard Nask. To match even the requirements of explicit segmentation, we add detailed, manual GT for all word samples, which includes bounding boxes of Pieces of Arabic Words (PAWs) and points where two letters are connected. Nevertheless the IESK-arDB word database contains only 300 different words (written by 20 writers), due to the time expensive GT. The manual, proper generation of such detailed GT for complete Arabic text pages is indeed more complicated and hard to realize even for few samples. To bypass this problem, it would be very helpful, if databases necessary for the research field could be produced automatically. One possible way to accomplish this task is to generate synthetic handwriting samples of single words and texts.

**Figure 1:**
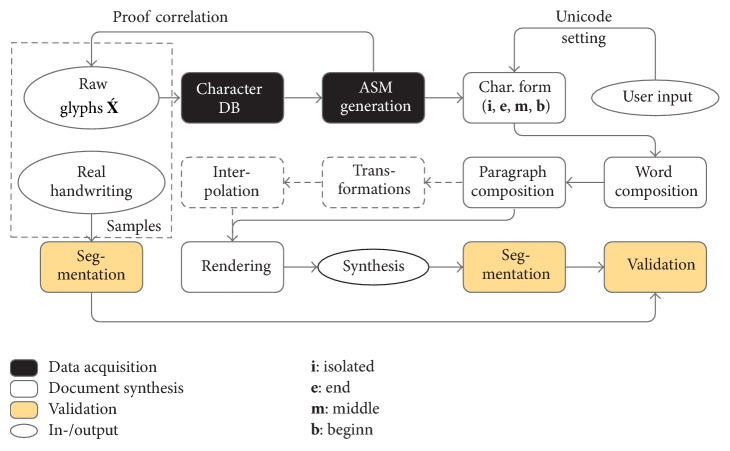
Simplified flowchart of the proposed synthesis system.

We developed a system for this purpose, which allows creating synthetic databases from text files or Unicode that is entered within the user interface (UI).  Figure 1 gives an overview of the design of that system. Ground truth are automatically generated. They contain original Unicode, ArabTeX transliteration, and further data as the bounding box of every letter. Furthermore the trajectories are stored for online applications. The system is capable of generating realistic synthesis of words, text lines, and complete (single column) text pages.

The rest of the paper is organized as follows. In Section 3 we give an overview of the related work. Thereafter we outline necessary data acquisition steps in Section 4. We also detail the mathematical background of Active Shape Models (ASMs) that we use to generate a large number of polygonal variations of Arabic letters. In Section 5 we describe our proposed methods to synthesize words and text pages by composing and arranging ASM based glyphs. Experimental results are discussed in Section 6, where we use synthesized databases to validate a segmentation method. Conclusion and future work are presented in the last section.

## 2. Arabic Script

The Arabic script has some special characteristics, so that synthesis or OCR approaches for Latin script will not succeed without major modifications [6]. Important aspects of Arabic script are as follows:

1. Arabic is written from right to left.
2. There are 28* letters* (Characters) in the Arabic alphabet, whose shapes are sensitive to their form (isolated, begin, middle, and end); see Table 1.
3. Six characters can only assume the isolated or end form, which splits a word into two or more parts, called* Piece of Arabic word* (PAW). They consist of the main body (connected component) and related diacritics (dots), supplements like Hamza (أ). In case of handwriting, the ascenders of the letters Kaf (ك), Taa (ط), or Dha (ظ) can also be written as fragments.
4. Arabic is semicursive: within a PAW, letters are joined to each other, whether being handwritten or printed.
5. Very often PAWs overlap each other, especially in handwritings.
6. Sometimes one letter is written beneath its predecessor, like Lam-Ya (لي) or Lam-Mim (لم), or it almost vanishes away when it is in middle form, like Lam-Mim-Mim (لمّ) (unlike the middle letter of Kaf-Mim-Mim (كم)). Hence, in addition to the four basic forms, there are also special forms, which can be seen as exceptions. Additional, there are a few ligatures, which are two following letters that build a completely new character like LamAlif (لا).
7. Some letters like Tha (ث), Ya (ي), or Jim (ج) have one to three dots above, under or within their “body.”
8. Some letters like Ba (ب), Ta (ت), and Tha (ث) only differ because of these dots.

## 3. Related Work

There are several applications of text synthesis, such as word spotting, CAPTCHAs, character recognition improvement, calligraphy, and others [2]. Accordingly there are different approaches of synthesis. In the literature, research addressing the issue of synthetic text generation can be classified into two main categories, top-down and bottom-up approaches. Both can be either based on offline or online techniques, according to the available samples and applications. Top-down approaches are typically based on neuromuscular models, that simulate the writing process itself [7, 8]. Therefore, script trajectories are seen as a result of character key points (built by the human brain when learning how to write), the writing speed, character size, and inertia, which finally leads to the curvature of handwritings. These approaches are focused more on the physical aspects of writing than the actual handwriting sample outcome. One typical application is to investigate diseases as Parkinson or Alzheimer that influence handwriting abilities [9].

Bottom-up approaches on the contrary model the shape (and possibly texture) of handwritings itself. Hence, bottom-up approaches are preferred in context of text recognition task like segmentation or handwriting recognition. Bottom-up approaches can be further categorized into generation of new samples of the same level and concatenation to more complex outcomes, such as words, that are composed from characters or glyphs [10].

A common generation technique is data perturbation, which is performed by adding noise to online or offline samples. Samples might be complete units as text lines or words, but single characters or glyphs (as syllables, ligatures, or modified letters) are used mostly [11]. In case of letters or glyphs, noise is often achieved by degradation of offline or online samples or random displacement of trajectories; transformations as shearing, scaling, or rotation are favored for perturbing words or text lines. Another generation technique is sample fusion that blends two or a couple of samples to produce new hybrid ones [12, 13]. A better statistical relevance can be achieved using model based generation [14, 15]. This initially requires the creation of deformable models, which represent a class by a flexible shape. Deformable models are often based on statistical information that must be extracted from sufficient samples (usually on character level). Then unlimited new representations of the same model class can be generated. At the same time, deformable models are capable of generating more realistic variances than data perturbation, which closely depict the peculiarities of the letter class. Examples for deformable models are Active Shape Models (ASMs) and novel Active Shape Structural Models (ASSMs), which are used for generating variances of simple drawings and signatures [16]. ASMs are also applied for the classification of Chinese letters [17].

Since documents in Latin based script might be handprinted, concatenation of such handwriting samples to units of higher levels can be done without connecting the samples [10]. Proper simulation of cursive handwriting requires at least partially connection though. There are approaches that connect offline samples directly [18] and those who use polynomial [19], spline [20, 21], or probabilistic models [22]. Due to the semi cursive style, connecting is mandatory in case of Arabic script.

Systems using the described techniques to synthesize handwritings have been built for different scripts and purposes. Wang et al. [23] proposed a learning based approach to synthesize cursive handwriting by combining shape and physical models. Thomas et al. [24] have proposed synthetic handwriting method, for generation of CAPTCHAs' (*completely automated public Turing test to tell computers and humans apart*) text lines. After segmentation, samples for each letter are generated using shape models. In the synthesis process, a delta log-normal function is used to compose smooth and natural cursive handwriting. Multiple approaches specific to Latin script that are based on polynomial merging functions and Bezier curves have been documented in [20]. Miyao and Maruyama [25] have proposed a method to improve offline Japanese Hiragana character classification using virtual examples synthesized from an online character database.

In contrast to word synthesizing, little research has been done concerning text line, paragraph, or document synthesis. If synthesis of multiple text lines is considered at all, it is typically modeled as horizontal baselines that may be influenced by noise [21], or each baseline line is defined by a rotation angle [10]. Varga et al. [26, 27] presented a method for handwritten English text line synthesizing; their methodology starts by composing static image of the text line by perturbing and chaining of character templates. Then text line is drawn using overlapping strokes and delta-lognormal velocity profiles, as stated by delta-lognormal theory. Chaudhuri and Kundu proposed a system to synthesize handwritten Bangla script pages [28]. For page layout simulation they compute Gaussian distributions from natural text pages to model different features, namely, left margin angle, line orientation, interline gap, and line undulation.

Due to the characteristics if Arabic script, which were discussed in Section 2, existing systems and methods can not be directly used for Arabic. Margner and Pechwitz [29] suggest an image based perturbation approach for the generation of synthetic printed Arabic words. They add global noise of different degrees, simulating degradation, as artifact, which are caused by repeated copying. As for the problem of automatic synthesis of offline handwritten Arabic text, to the best of our knowledge, Elarian et al. [3, 30] are the first who published research work addressing this problem so far. They propose a straightforward approach to compose arbitrary Arabic words. The approach starts by generating a finite set of letter images from two different writers, manually segmented from IFN/ENIT database, and then two kinds of simple features (width and direction feature) are extracted, so they can be used later as a metrics in the concatenation step. Saabni and El-Sana proposed a system to synthesize Pieces of Arabic Words (PAW) (without diacritics) [31]. They use digital tablets to acquire online samples, which are randomly composed to PAWs. Thereafter a subset of the produced synthetic data is selected by clustering techniques to get a compact database. In [32] we proposed a system to generate Arabic letter shapes by ASMs built from offline samples. Subsequently, we developed a system to render images of Arabic handwritten words, concatenating ASM based samples and using transformations on word level as optional, second generation step [33].

Segmentation of Arabic words is quite challenging. It depends on holistic word as well as the features of single characters, and detailed GT are required to perform a useful validation. Hence, it is reasonable to test this method on synthetic databases, which contain such GT. One of the earliest segmentation based approaches, suggested for the recognition of Arabic handwritten text, is the one proposed by [34], and no segmentation results are reported though. Xiu et al. proposed probabilistic segmentation model, for this segmentation approach a tentative, contour based over segmentation is first performed on the text image; as a result, a set of what they called graphemes is produced [35]. The approach differentiates among three types of graphemes. The confidence of each character is calculated according to the probabilistic model, respecting other factors, for example, recognition output, geometric confidence, and logical constraint. The authors experimented the proposed methodology on five different test sets, achieving 59.2% success rate.

## 4. Data Acquisition and Generation

To synthesize Arabic handwritten words from glyphs, a sufficient amount of* glyph samples* has to be acquired first. In our case trajectories of single letters and their connections (Kashidas) are used as glyphs. The glyphs are acquired with online techniques since relevant information can be extracted more efficiently from trajectories than images. Nevertheless, we are mainly interested in synthesizing offline data. Hence, we decided to use online pens for data acquisition, for they can be applied as ordinary biros in contrast to digital tablets. Fifty or more samples per writer are taken from over hundred letter classes (28046 samples altogether) to built an online* character database*. To minimize manual effort and allow an easy extension, this database is completely automatically generated from raw data. Raw data are trajectories $\overset{´}{X}\in {\mathbb{R}}^{m\times 2}$ (see Table 2) for each stroke within a virtual DIN A4 page. That page has a resolution of 1000 dpi and 58 rps (reports per second), so there are constant timestamps $\overset{´}{▵t}=t_{i+1}-t_{i}=0.0172\;s$ between neighbored $({\overset{´}{x}}_{i,1},{\overset{´}{x}}_{i,2})\in \overset{´}{X}$. The generated database contains the trajectories and an image representation for each Arabic letter class (see Table 1), as well as the resulting Active Shape Models (ASMs), which are described in the next section. Digits and special characters might be added in future work.

### 4.1. Computing Active Shape Models for Generation of Arabic Characters

Active Shape Models (ASMs) are statistical representations of the approximated shape of an object. An ASM uses the distribution of some significant points to store the most important information of many shapes of a class in one single model. Normally some well-defined landmarks have to be set manually for all samples and additional intermediate points between these landmarks are used if there are not enough landmarks to represent the shape. However, the definition of landmarks for over hundred classes for every writer is barely realizable and would prevent to add data from further writers efficiently. This is why we use two given landmarks, the* Start* and the* End* point of each polygon, and compute intermediate points by interpolating between the given $\left({\overset{´}{x}}_{i,1},{\overset{´}{x}}_{i,2}\right)$ of $\overset{´}{X}\in {\mathbb{R}}^{m\times 2}$ to get polygons **X** ∈ *ℝ* ^*n*×2^. By alternate storing of the *x*_*i*,1_ and *x*_*i*,2_ coordinates, each polygon is represented as a single vector of size 2*n*: **x** = (*x*_1,1_, *x*_1,2_, *x*_2,1_, *x*_2,2_ …, *x*_*n*,1_, *x*_*n*,2_)^*T*^ that is required to build ASMs. Given the point numbers *m* of the original and *n* of the desired approximated samples, we interpolate in such a way that the time steps ${▵}_{t}=\overset{´}{{▵}_{t}}m/n$ between neighbored interpolated points **p**_*j*_, **p**_*j*+1_∣**p**_*j*_ = (*x*_2*j*_, *x*_2*j*+1_) are still constant, the Euclidean distance however may vary. Given the original points $\overset{´}{p}\in \overset{´}{X}$ we compute an interpolated point

**Figure 2:**
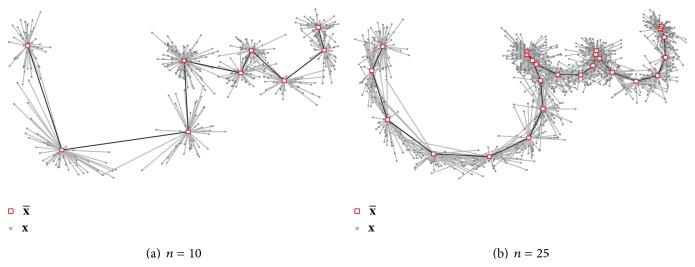
The average $\bar{x}$ and *r* samples **x** for the letter Sin in isolated form of writer 1. (a) The number of interpolation steps *n* = 10 is not sufficient to represent complex characters as Sin (س), and details will be lost or distorted. (b) With *n* = 25, the approximated samples **x** are reliable enough to build proper ASMs for all character classes.

**Figure 3:**
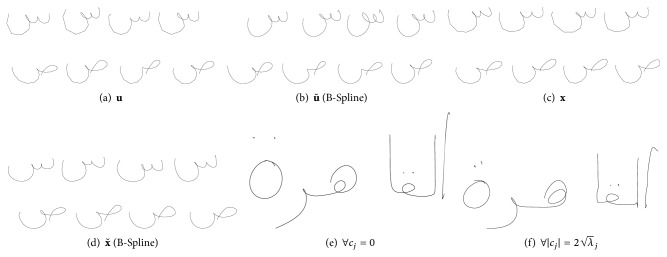
Examples of glyphs: (a) examples for ASM-glyph representations **u** (where $\sum \left|c_{j}\right|\approx \sum 1.6{\sqrt{\lambda }}_{j}$; see (3)), (b) a B-Spline interpolation has been applied on the representations, (c), (d), original letter samples **x**. A B-Spline interpolation with 5 steps has been applied on (b) and (e). Examples of ASM-glyphs **u** that are composed to words are shown in (e) and (f), where the influence is minimal (e) and maximal (f), respectively, for all eigenvalues.

(1) pj=1−λap´j′+λap´j′+1with  j′=j▵t~,  ▵t~=▵t´▵t,  λa=j▵t~−j′,

where *j*′ ∈ {1, *m*} and *j* ∈ {1, *n*}. This enables a more detailed modeling of complex structures as the peaks of Sin (س) as one can see in Figures 2 and 3. We set *n* = 25, which is sufficient to represent even complex Arabic characters like Sin (س), as shown in Figure 2 (in order to speed up the synthesis process, *n* could be optimized for each individual class).

We then scale all samples **x** keeping their aspect ratio. Let *W* be the width and let *H* be the heights of an unscaled **x**, and then we scale **x** by 100*x*100/(*H∗W*) and translate **x** so that its center lies within the origin.

For each class there are *r* available vectors, from which the ASM of the corresponding class is calculated (we set *r* = 50 for our experiments).

To build ASMs, the expected value $\bar{x}$ and the covariance matrix **S** have to be calculated first:

(2) x¯=∑i=1rxir,S=12r−1∑i=1rxi−x¯xi−x¯T.

As a consequence of the covariance matrix calculation, the Eigenvalues *λ*_*i*_ and Eigenvectors **e**_*i*_ can be determined, and then the ASM corresponding to character classes is calculated. Now any arbitrary number of vectors that represent each class can be calculated by linear combination:

(3) $$\begin{array}{c} u=\bar{x}+\overset{r}{\underset{j=1}{\sum }}c_{j}e_{j},\;c_{j}\in \left[-2{\sqrt{\lambda }}_{j},2{\sqrt{\lambda }}_{j}\right]. \end{array}$$

A limitation of *c*_*j*_ by $\pm 2{\sqrt{\lambda }}_{j}$ assures that all the deviations of **u** are within the doubled standard deviation *σ*. This is a common limit, since most training samples lie within this range. In few cases a limit of maximal $\pm 3{\sqrt{\lambda }}_{j}$ is applied, which requires a clear increase of *r* in order to keep **u** statistically reliable.

Some examples of **x** and **u** are shown in Figure 3. The computation of ASMs is quite costly, especially if many samples and interpolation points are used. This is why we avoid recalculations of ASMs at runtime, since the synthesizing process requires a minimum of 100 ASMs. Hence, all ASMs (including their corresponding online samples) are saved in files, to separate ASM generation from the synthesis module.

### 4.2. ASM Distance Measure

The distance *δ* of a sample **x** and a representation **u** of an ASM can be calculated as follows:

(4) $$\begin{array}{c} \delta \left(\;x,u\;\right)=\frac{1}{n}\overset{n}{\underset{i=1}{\sum }}\sqrt{{\left(u_{2i-1}-x_{2i-1}\right)}^{2}+{\left(u_{2i}-x_{2i}\right)}^{2}}. \end{array}$$

**Figure 4:**
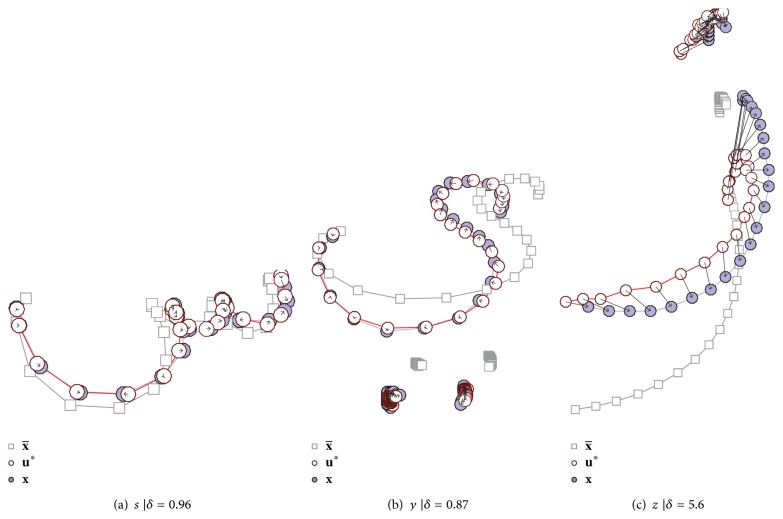
Examples of ASM based glyph **u** ^*∗*^ that has the highest correlation (deviation *δ*) with a randomly chosen samples **x**. The average sample polygon $\bar{x}$ is displayed to show how strong **x** differs from the expected shape. Some of the ASM for characters with diacritics are problematic, as shown in (c).

Due to the performed scaling of **x**, *δ*(**x**, **u**) can be interpreted as the approximated deviation in percent. For all samples **x** we compute the most similar representation **u** ^*∗*^ by solving (3) numerically, using **c** as unknown (initially ∀*c*_*i*_ = 0). Some examples of **u** ^*∗*^ are visualized in Figure 4.

In contrast to ASMs from offline samples [32], most online based ASMs are capable of representing their input samples well, even without manually defined landmarks, since detailed, slowly written structures (where landmarks are suspected) are represented by more intermediate points. This effect allows an efficient extension and maintenance of the* character database*, since time consuming, manual landmarking can be avoided.

Nonetheless the average $\bar{\delta }$ is writer dependent. We measured $\bar{\delta }=0.8$ for writer 1, $\bar{\delta }=1.46$ for writer 2 and even higher values for the other writers, whose handwriting style is less tidy. Furthermore $\bar{\delta }$ depends on the letter class. A high $\bar{\delta }$ is mainly caused by some classes with diacritics as Zai (ز) that need improvement (see Figure 3(c)). The distance between the letters main body and diacritics can vary clearly, which may lead to inappropriate **u** ^*∗*^, in case that **x** is unlike $\bar{x}$. To react against this effect, more training samples could be used or diacritics and main bodies could be separated using Active Shape Structural Models (ASSM) [16]. However, our ASMs are meant for synthesis approaches where the use of pure noise (perturbed data) is quite common, and hence moderate imprecision does not spoil the synthesis quality significantly.

## 5. Synthesizing Arabic Handwritings

In the following subsections we describe all methods that are applied to create synthetic Arabic handwritings from Unicode, using the* ASM data* from the last Section. Our system uses these either to show a few specific syntheses for preview purpose or to generate a complete database including ground truth.

The methods, which are used to synthesize and render Arabic handwritings, are described in the following Sections.

### 5.1. Composing Words

The basic idea of Arabic handwriting synthesis from Unicode is to select glyphs with proper shapes (isolated, initial, end, or middle form) and connect them subsequently to build PAWs, words, and texts, from which images or vector graphics are rendered.

#### 5.1.1. Calculation of the Letter Form

Our system receives text as Unicode string that represents every letter as number. Since our samples are limited to the 28 regular letters and Tamarbuta (ة), we substitute special characters as Alif with Hamza above (أ) with their regular form Alif (ا) before starting the synthesis. Letter forms have a strong influence on the letter shape, but they are not given by regular Unicode; thus they have to be determined first.

Letters of the set Γ^2^ = (ادرزذو) can only assume** i**solated or** e**nd form. All other letters, such as Ayn() can assume begin-, middle-, and- as well as isolated- form. Ayn (عـ ـعـ ـع ع). Therefore, the form **f**_0_ of the first letter *𝔩*_0_ of a word is

(5) $$\begin{array}{c} f_{0}=\left\{\begin{array}{cc} i & \text{if }l_{0}\in \Gamma^{2}\\ b & \text{else}. \end{array}\right. \end{array}$$

**Figure 5:**
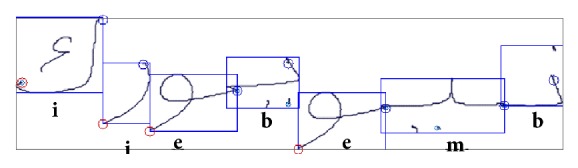
Outgoing from the Unicode sequence (ن ي و ي و ر ك) the forms (**i**solated,** e**nd,** m**iddle, and** b**egin) of all letters are determined (نـ ـيـ و يـ و ر ك) and composed to the word نيويورك (New York).

Examples of the different letter forms within an Arabic word are shown in Figure 5. Let **f**_−1_ be the form of *𝔩*_−1_, which is the predecessor of letter *𝔩*, and *𝔩*_+1_ = *⌀* define that the successor of *𝔩* is a space, tab, or return token *⌀*, and then the position **f** of a letter *𝔩* can be defined as follows:

(6) $$\begin{array}{c} f=\left\{\begin{array}{cc} i & \text{if}\;\;f_{-1}\in \left(i,e\right)\wedge \left(l\in \Gamma^{2}\vee l_{+1}=⌀\right)\\ b & \text{if}\;\;f_{-1}\in \left(i,e\right)\wedge \left(l\notin \Gamma^{2}\wedge l_{+1}\neq ⌀\right)\\ m & \text{if}\;\;f_{-1}\in \left(b,m\right)\wedge \left(l\notin \Gamma^{2}\wedge l_{+1}\neq ⌀\right)\\ e & \text{if}\;\;f_{-1}\in \left(b,m\right)\wedge \left(l\in \Gamma^{2}\vee l_{+1}=⌀\right). \end{array}\right. \end{array}$$

Letters that have only two forms split an Arabic word into Pieces of Arabic Words (PAWs), which consist of one or more letters.

#### 5.1.2. Selection of Suitable Glyphs

To ensure that the styles of all neighbored glyphs are similar, glyphs of different writer are not mixed within a synthesis. We encouraged the writers to write letters only in this style that is dominant for their writings and avoid severe rotations. The size of the glyphs is normalized by the average character size we extracted from the IESK-arDB. However, we corrected the size manually in case of rare character classes. A suitability measure for the glyph joint points has not been considered, since steady joints are achieved by B-Spline interpolation and rendering at the end of the synthesis process.

#### 5.1.3. Connecting Glyphs

After all letter classes are defined by their names and forms, corresponding ASMs are loaded. The ASMs are used to generate an unique polygonal representation **u** for each occurrence of a letter class in order to avoid piecewise identical syntheses. In order to compose words from these polygons, each letter in end or middle form has to be connected with its predecessor: عـ ر→عر, نـ ـيـ ـو→نيو. Let **p**_**f**_ be the first point of ـيـ and **p**_**l**_ the last point of its predecessor نـ, and then we can connect them by translating ـيـ by **p**_**l**_ − **p**_**f**_. An example is shown in Figure 5.

The relation of a PAW's *y* coordinate and the baseline depends on the letter classes the PAW is composed of. Thus we extracted the average *μ*_*r*_ and variance *σ*_*r*_ of the relative distance between the baseline and the center of a letter from manual created ground truth of our (real) word database IESK-arDB [4]. We set the baseline to 0 and shift all *y* coordinates of each PAW by *𝒩*(*μ*_*r*_, *σ*_*r*_). Finally, the space between the rightmost point of a PAW and the leftmost point of its predecessor has to be defined. Therefore, the user depending parameter *ς* is used that may be negative in order to simulate overlapping PAWs. Given **p**_**l**_ and **p**_**f**_, the PAW can be translated by the vector

(7) $$\begin{array}{c} t_{\text{paw}}=\left(\begin{array}{c} p_{l_{1}}-p_{f_{1}}-\varsigma \\ N\left(\mu_{r},\sigma_{r}\right) \end{array}\right). \end{array}$$

In case of intersections between the polygons of overlapping PAWs, *ς* is increased iteratively by 25% of the average letter width, as described in Algorithm 1.

**Figure 6:**
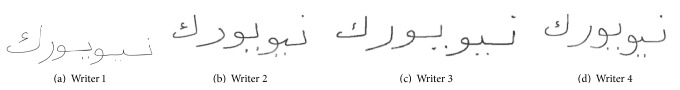
The word نيويورك (New York), synthesized by the average letter shapes $\bar{x}$ of different writers.

Examples of words composed by the average letter shapes of different writers are shown in Figure 6. Examples from ASM based and original samples can be found in Table 3. The maximal deviation *σ*_*A*_ means that the influence of an eigenvector **e**_*i*_ is limited by $\pm 2\sqrt{\lambda_{i}}$. While letters look similar using *σ* between 0 and 1, ASM representations **u** with *σ*_*A*_ of 2 already provoke increased letter variation. ASMs (trained with *r* = 50 samples) are barely capable of representing a deviation of *σ*_*A*_ = 3 though. As a matter of fact, there are noise based deformations as shown in Table 3 (second last row). This effect might be intended to create especially challenging syntheses.

### 5.2. Simulation of Global Variances

**Figure 7:**
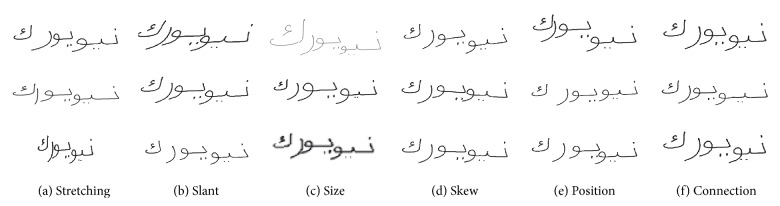
Affine transformations and connection shortening on synthetic words.

ASMs already contain variations in slant, width or connection size. Nevertheless, these variations are limited by the used samples. In order to increase and control these variations, affine transformations are used (scaling, translation, shearing, and rotation), which allow optional manipulations of letter and PAW shapes. The user interface (UI) of our synthesis system allows to set the average *μ* and variance *σ* of a Gaussian distribution for all affine transformations. Particularly global variations as the slant can be achieved this way. The influences of these affine transformations on the resulting word image is shown in Figure 7.

A stretching is performed by scale each *x* component of each letter point by the factor *c* ∈ [0.5,2]. The word slant can be set by shearing the word with an angle of *α*_*s*_ as the skew of PAW can be manipulated by rotating to the angle *α*_*r*_, where *α*_*s*_, *α*_*r*_ ∈ [−45°, +45°]. We analyzed the skew and slant of samples of the IESK-arDB database [4] with local minima regression and Hough transform. We found that the skew correlates with a Gaussian distribution of *𝒩*(−3.8°, 7.1°) (passed Chi square test with *α* = 0.05). The slant can be represented by *𝒩*(−4.6°, 14.4°). The size of the complete word or single letters can be adjusted by an equal scaling with *c* ∈ [0.1,10], which can be used to control the resolution of the synthesized word images or to increase variation of letter size. As described in Section 5.1.3 variations of PAW positions can be realized by translations. Even using the same glyphs, synthetic words can assume large variations in shape when using affine transformations or cutting connection size, as shown in Figure 7.

**Figure 8:**
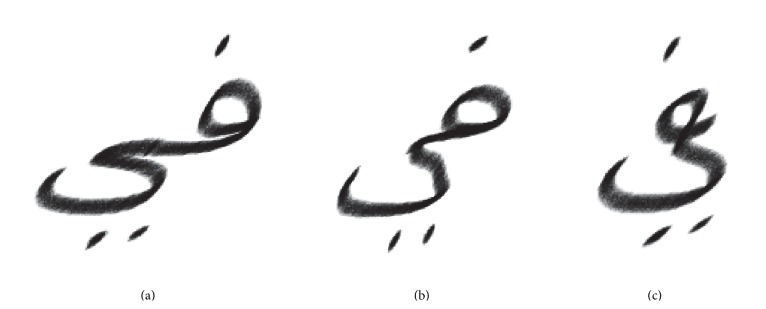
Example for a synthesized letter pair using (a) full (b) half and (c) negative* Kashida* length.

When examining the glyph samples and synthesis we found that compared to complete handwritings, letter connections of the acquired samples are extended and even excessively (approximately twice as) long in case of writer 1. Hence, we allow to delete up to 25% of the letter points to simulate stretched or missed connections, which often occur in natural Arabic handwritings. In Arabic handwritings some characters as Ya (ي) are sometimes written beneath their predecessors. As a result they resemble a single character, which impedes segmentation and recognition tasks. This effect can be simulated by a strong reduction of the Kashida, as shown in Figure 8. However, this feature has been not yet entirely implemented, since a list of all pairs of letters that typically show this behaviour needs to be created first.

### 5.3. Interpolation

Since a polygonal letter representation does not look natural for vector graphics or images with a scaling factor >1, we use interpolation to improve the outcome. Let *n*(**u**) be the number of points of **u**, and then we use **u** as control points to interpolate a curve $\overset{˘}{u}$. By increasing the interpolation steps, $n(\overset{˘}{u})$ can be approximated to the original number of points of a sample $\overset{´}{X}$. The average length of $\overset{´}{X}$ is $\bar{n}(\overset{´}{X})=266.8$, and hence a tenfold increase $n(u)=25\to n(\overset{˘}{u})=250$ is generally sufficient to achieve smooth syntheses. To ensure that the above mentioned methods work efficiently on the compressed representation **u**, the interpolation step is applied just before rendering or skipped in case of low-resolution synthesis.

**Figure 9:**
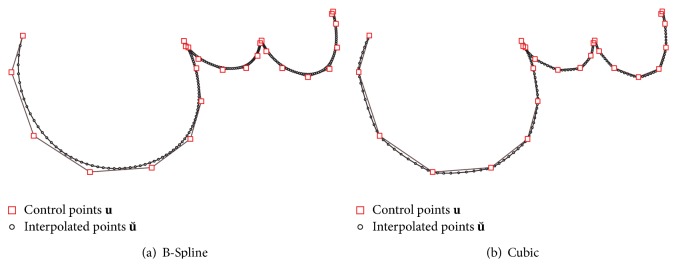
Comparison of B-Spline and Cubic interpolation where *n*(**u**) = 25 and $n(\overset{˘}{u})=250$.

Our system supports two interpolation methods. Piecewise Cubic hermite-interpolation is *C* ^1^-steady, which means that only the first derivation of the used interpolation function is steady. Therefore, hermite-interpolation leads to less smooth and accurate results, a property, that can be used to create noisy handwritings [32] (perturbed data technique). State-of-the-art B-Spline interpolation is commonly used within CAD-applications, since it is *C* ^2^-steady and leads to smooth, natural curves, which can be defined properly by their control points, as shown in Figure 9. Although the B-Spline curve do not pass through all control points, it fits the original curve sufficiently when *n* = 25 control points are used. Given the measure *δ* of (4), the average distance between **u** and the *n* closest points of $\overset{˘}{u}$ can be computed. Using a cross-validation with *k*fold = 10, we get $\bar{\delta }(u,\overset{˘}{u})=0.45$ with a variance of 0.058.

### 5.4. Rendering Handwriting Images

The former sections discussed how to compose and interpolate polygonal representations of Arabic handwritings. Now those have to be transformed into images and saved in common files formats (.bmp,  .png, etc.), which can easily be loaded by most text recognitions or document analysis systems. Even preprocessing steps, as thinning, can influence the performance of the following tasks. Such preprocessing is sensitive to secondary features or flaws, which are a result of the used writing materials; hence the synthesized files should contain those features too. Therefore, we propose a rendering technique that reflects optical features caused by common pens as ball pens or pencils [33]. Subsequently, a modification of this technique is described that allows rendering features of historical handwritings.

**Figure 10:**
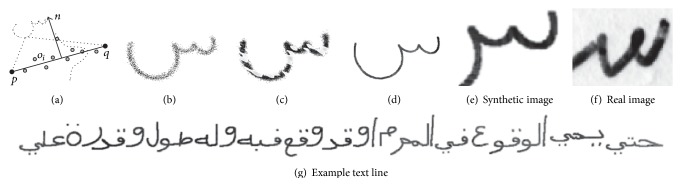
Painting technique: (a) scheme, (b) word shape with *ρ* = 6, *σ* = 3.5 (Gaussian filter is disabled), (c-d) example results for painting technique: (c) test texture applied on (b), (d) *ρ* = 30, *σ* = 1.0 with ball pen texture, and (e-f) comparison of natural and synthetic ball pen textures.

First of all, pixels **o** have to be found that are close to polygons $\overset{˘}{u}$ and belong to the foreground. As shown in Figure 10(a), we first interpolate between two neighbored points **p**, **q** of $\overset{˘}{u}$ (or **u**) and get *ρ∗*‖**p** − **q**‖ new points

(8) $$\begin{array}{c} o^{\prime }=\nu p+\left(\;1-\nu \;\right)q,\;\nu \in \left[0,1\right], \end{array}$$

where *ν* is uniform distributed and *ρ* is a user defined parameter.

Let $\vec{n}$ be the normal vector of line $\bar{pq}$ and then we shift each **o**′ along $\vec{n}$ using a Gaussian distribution

(9) $$\begin{array}{c} o=o^{\prime }+N\left(\;\mu ,\sigma \;\right)\vec{n}, \end{array}$$

where *σ* and *μ* define the line width, which is decreased up to 20%, in case that **p** lies between the* Pen Up* or* Pen Down* point and the neighbored control point. As a result, the prototype of a word image has been prepared that defines all pixels, which are influenced by pigments at all (shown in Figure 10(b)). To allow sharp contours, a limitation of |*𝒩*(*μ*, *σ*)| < *ϖσ* is applied according to the simulated pen, where *ϖ* is user dependent.

#### 5.4.1. Texture

Biros or pencils cause an irregular pigmentation intensity, which is reflected by pixel intensities *I*(*x*, *y*) of scanned images. A realistic physical model that simulates this behavior in a proper way is beyond the scope of this paper; a more simplified and generalized approach however is quite practical. Inspired by the ability of Fourier transform based image compression to represent the main nature of a texture by a small subset of the underlying frequencies, we define a texture by 10 points (*λ*, *θ*, *ϕ*) in frequency space in order to emulate irregularities caused by pen and paper. This way, simplified but unique textures can be created at runtime. However, in contrast to image compression, we are not interested in avoiding noise-prone high frequencies. Hence, different high and low frequencies are combined to simulate regional as well as locale effects (behavior of ink, paper texture). Each point represents a texture layer in image space with *I*_1_(*x*, *y*)∈[−1,1]. We created several texture classes by defining different sets of Gaussian distributions for the angle *θ*, wave length *λ*, and phase shift *ϕ* of all layers manually. Afterwards, pixel intensities *I*_2_(**o**)∈[0,256] are computed by accumulating most texture layers. The left layers are used to achieve variations in their intensities by multiplication. Finally, an image **I** with a small margin is created and all **o** are fitted to **I** subsequently.

By creating word syntheses using polygonal glyphs and rendering techniques, smooth letter connections can be achieved more efficiently compared to synthesis approaches that use image based glyphs. Furthermore, in contrast to the usage of natural textures, the described technique is able to generate unique, nontiled textures for every synthesized word, as shown in Figures 10(c)–10(e). Depending on the texture class, a median or Gaussian filter is used before saving the image.

#### 5.4.2. Simulation of Feather Like Writing Tools

The previous method allows a proper simulation of text that is written by ball pens, pencils, or coal on white paper. However, to allow a more accurate simulation of writing instruments as fountains pens or feathers, we extended our rendering technique. This includes mainly the implementation of two features: the writing speed and the shape of the top of the writing instrument, further called* Pen Shape*.

*Pen Shape.* If the* Pen Shape* is modeled as line or ellipsoid, the line width of the trajectory $\overset{˘}{u}$ depends not only on the width of the* Pen Shape w*_*P*_, but also on its angle *γ*_*P*_ and the angle of the trajectory tangent $\gamma_{\overset{˘}{u}}$. We initialize *γ*_*P*_ = 45°, changing it continuously with a maximal deviation of ±15°. This locale deviation is computed by adding two cosine functions, where the wavelengths, phases, and amplitudes are redefined by Gaussian distributions for each synthesis.

**Figure 11:**
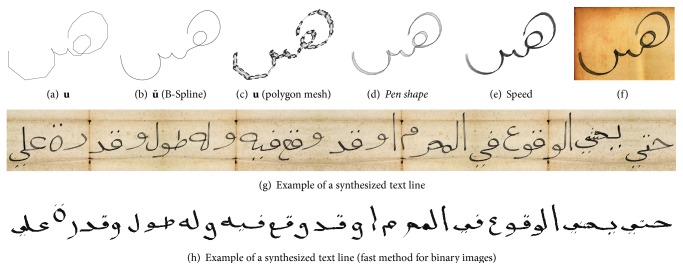
From the polygon **u** to a synthetic image that simulates a feather like writing instrument. (c) Polygon mesh created from **u** that is used for fast rendering of binary images.

According to the texture of a* Pen Shape*, the contact with the paper and consequently the caused pigmentation can vary. This is simulated by an one-dimensional function *f*(*x*_*P*_)∣0 ≤ *x*_*P*_ ≤ *w*_*P*_ that defines the pigmentation potential for the long axis of the* Pen Shape*. An emphasized example, inspired by a fountain pen, is shown in Figure 11(d).

Binary images can be rendered in a faster way by defining polygons (here a triangle mesh) that is a result of extruding an one-dimensional* Pen Shape* along **u** or $\overset{˘}{u}$. A visualization of this can be found in Figure 11(c), and a resulting synthesis is shown in Figure 11(h). These polygons can then be drawn by standard routines. If the angle of the Pen Shape and the line is identical or so close, where the polygon width would be smaller than one pixel, a line have to be drawn instead to avoid letter fragmentation.

*Writing Speed.* Large lines or bows, as the left part of Sin (س), are usually written faster than more complex structures. A high writing speed often causes a lack of pigmentation that leads to brighter or dappled lines. However, there is no need to reconstruct writing speed, for the used online letter samples already that contain such information. From each representation $\overset{˘}{u}$ we can extract the relative, local writing speed for ASMs representations in points per second

(10) vi pt/s=u˘2i−1,u˘2i−u˘2i+1,u˘2i+2 ptnu/nu˘Δt s,1≤i≤nu˘.

In order to estimate the expected pigmentation intensity, we use the normalized writing speed $\dot{v}\in [0,1]$, where $\dot{v}=0$ is the slowest and $\dot{v}=1$ is the fastest part of the trajectory of a synthesis. If *𝔠*_0_ is the current color of a pixel in RGB color space and *𝔠*_*p*_ is the color of the pen pigment, then the new color is computed by

(11) $$\begin{array}{c} c=c_{p}\alpha_{a}+c_{0}\alpha_{b}\left(\;1-\alpha_{a}\;\right),\;c\in R^{3}, \end{array}$$

where $\alpha_{a}=0.8(1-\dot{v})+0.2$ defines opacity of *𝔠*_*p*_ and *α*_*b*_ = 1 defines the opacity of the background. Although pigments that are used for handwritings are typically full or semiopaque, reduction of pigmentation caused by increased writing speed can lead to transparency. This is simulated by reducing *α*_*a*_, as shown in Figure 11(e).

To ensure a steady behavior, we interpolate the speed of connected letters. For the first $n(\overset{˘}{u})/4$ points $\left({\overset{˘}{u}}_{2i-1},{\overset{˘}{u}}_{2i}\right)$ we set the speed $\dot{v}$ of a letter in middle or end form to

(12) v˙i≔v˙i1−λi+v˙bnu˘λi,λi=4inu˘,  i≤14nu˘,

where ${\dot{v}}_{b}(n(\overset{˘}{u}))$ is the speed at the end of the previous letter. The effect on the synthesis is shown in Figure 11(d).

*Render on Degraded Background*. Finally, we create Figure 11(f) by combining our texture rendering technique of Section 5.4.1 with the one based on* Pen Shape* and writing speed, using a nonuniform background. Therefore, we applied a transparent texture on all pixels that does not belong to the background texture. This way, small, random irregularity is simulated.

In the shown examples a black color is used, as most documents are written with dark ink (like iron oxide or soot). Pigmentation intensity is implemented as transparency, which allows simulating pigment accumulation at crossing lines or in case of a textured background. However, also colored opaque or semiopaque ink can be simulated. This might be interesting in the context of historical documents, since important passages are often highlighted by using red ink. Another important feature of historical documents is degradation of paper or parchment. Currently we simply use images of natural paper or parchment as background textures, which are scaled or tiled in case where they are smaller than the synthesized document.

### 5.5. Generation of Text Pages

Recently, not only character or word recognition but also more complex document analysis issues that address the interpretation or recognition of complete documents become a focus of Arabic handwriting research. Hence, we investigate the possibilities of text page synthesis. In this regard the accurate simulation of the lower baselines is crucial. Many problems that occur while detecting lines, words, or connected components depend on these baselines, as for instance assigning diacritics (that are very close to more than one text lines) to their corresponding PAW.

Our approach of simulating baselines has three steps. First of all we set the coordinates (*x*_0_, *y*_0_) of the first letter for each line. Secondly the curvatures of the baselines have to be computed. Thirdly potential intersection of words have to be solved.

*Start PAWs*. Due to the style of Arabic handwritings, all lines start at the rightmost *x*-coordinate *x*_0_ = *x*_max⁡_. The vertical space between two neighbored lines can be defined as percentage of the average PAW heights to get *y*_0_ for each baseline.

*Baselines*. We implement the second step by declaring functions *f*(*x*, *y*_0_) that define the curvatures of the simulated Baselines Ξ_*s*_ (*y*_0_ defines the initial heights of a line). We normalize $\overset{´}{y_{0}},\overset{´}{x}\in [0,1]$ inside *f*(*x*, *y*_0_), so it becomes independent of the page size. How to compute proper *f*_*i*_ is explained in Section 5.6.

**Figure 12:**
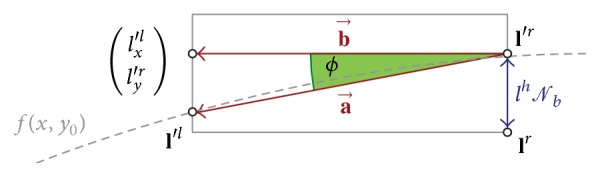
Arranging a letter along the baseline.

Let **l** ^*r*^ define the lower right of a letters bounding box, then we first translate the letter **l** towards the baseline, so that **l** ^*r*^ touches it. Subsequently, the *y*-position of **l** must be corrected according to its class by translating it by **t**_*b*_, so that **l** ^*r*^ might be above or below *f*(*l*_*x*_^*r*^, *y*_0_), as shown in Figure 12. Therefore, we extracted the normalized statistical relation *𝒩*_*b*_ between **l** ^*r*^ and the baseline from the IESK-arDB for all letter classes. If *l* ^*h*^ is the heights and **l** ^*i*^ is the *i*th point of **l**, we can compute **t**_*b*_ = (0, *l* ^*h*^ *𝒩*_*b*_)^*T*^ and **l** ^′*i*^ = **l** ^*i*^ + **t**_*b*_ as shown in Figure 12. We perform this for every first letter **l**_*f*_ of a PAW. Apart from the position, also the PAW skew depends on the baseline. We simulate this by rotating all points **l** ^′*i*^ of a letter around their* pen down* point **l** ^′1^. Let **l** ^*l*^ be the lower left of the bounding box of the current letter **l**, and then the rotation angle is

(13) $$\begin{array}{c} \phi =\cos^{-1}\frac{\langle \vec{a},\vec{b}\rangle }{\left|\vec{a}\right|\left|\vec{b}\right|},\;\vec{a}=l^{\prime r}-l^{\prime l},\;\;\vec{b}=l^{\prime r}-\left(\begin{array}{c} l_{x}^{\prime l}\\ l_{y}^{\prime r} \end{array}\right). \end{array}$$

Before we rotate a letter **l**′ ≠ **l**_*f*_′ it has to be connected with its predecessor **l**_*p*_′ translating it by **t**_*j*_ = (**l**_*p*_^′*e*^ − **l** ^′1^). This way, the handwriting synthesis fits to the baseline without causing aliasing effects.

*Solving Intersections.* In the last step, we detect and solve intersections between lines. Therefore, all PAW *𝔅* of the line above have to be detected, whose bounding boxes overlap with the current PAW *𝔄* or whose distances are less than *ϵ*. For all *𝔅* we then calculate weather there is any intersections between line segments ${𝔰}_{𝔄}=\bar{l_{𝔄}^{\prime i}l_{𝔄}^{\prime i+1}}\in 𝔄$ and ${𝔰}_{𝔅}={\bar{l_{𝔅}^{\prime i}l^{\prime i+1}}}_{𝔅}\in 𝔅$. If so, *𝔄* is translated by (0, *ψ*)^*T*^. This has to be done also for the predecessor of *𝔄*, by using the translation (*ψ*, 0)^*T*^. Similar to Algorithm 1 (where intersections of two neighbored PAWs of the same line are handled) these steps have to be repeated, until no intersection is detected anymore.

**Figure 13:**
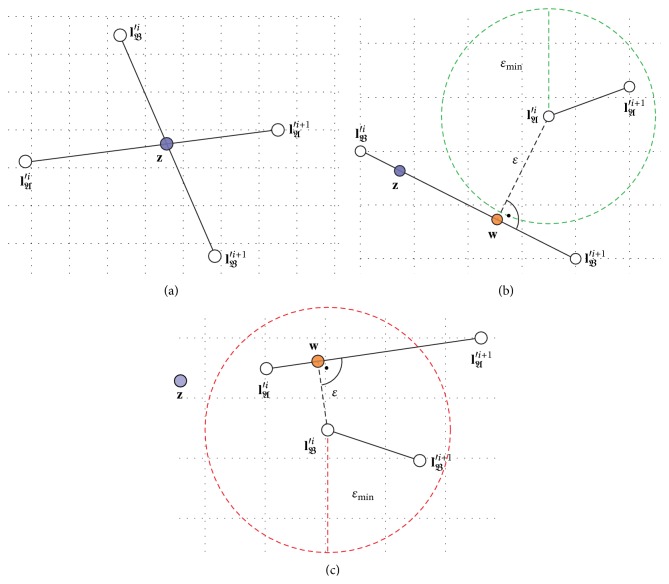
Detecting intersections of two line segments, where the grid has the size 1 pt to suggest the synthesized image, *ɛ* is 2 pt. (a) The lines segments intersect. (b) The line segments are close and *𝔰*_*𝔅*_ points to *𝔰*_*𝔅*_, but there is no need to move *𝔄* since their shortest distance *ɛ* is higher than *ε*_min⁡_. (c) The intersection point **z** is outside both line segments, but *ɛ* is smaller than *ε*_min⁡_, so an intersection of the corresponding lines of the synthesized image is expected.

Two lines, which are not parallel, have an intersection point **z**. To proof weather two line segments intersect, **z** of their corresponding lines must be calculated first. A pair of line segments *𝔰*_*𝔄*_ and *𝔰*_*𝔅*_ intersects, if and only if **z** lies on both line segments, as shown in Figure 13(a). However, there might be an intersection of lines in the rendered image, even if *𝔰*_*𝔄*_ and *𝔰*_*𝔅*_ do not intersect. To avoid this, the distance *ɛ* of the two line segments has to be higher than *ε*_min⁡_, where *ε*_min⁡_ has to be at least one pt larger than 2 times the* Pen Shape* widths *w*_*P*_. Let **w** be the point of the opposite line segment of **l**_*ɛ*_ ∈ {**l**_*𝔄*_^′*i*^, **l**_*𝔄*_^′*i*+1^, **l**_*𝔅*_^′*i*^, **l**_*𝔅*_^′*i*+1^} that is closest to **l**_*ɛ*_, and then we get *ɛ* = ‖**l**_*ɛ*_ − **w**‖. In case that **z** lies only on one line segment *𝔰*_*𝔅*/*𝔄*_, we know that **l**_*ɛ*_ ∈ {**l**_*𝔄*/*𝔅*_^′*i*^, **l**_*𝔄*/*𝔅*_^′*i*+1^}, as shown in Figure 13(b). Otherwise ‖**l**_*ɛ*_ − **w**‖ must be calculated for all four points.

**Figure 14:**
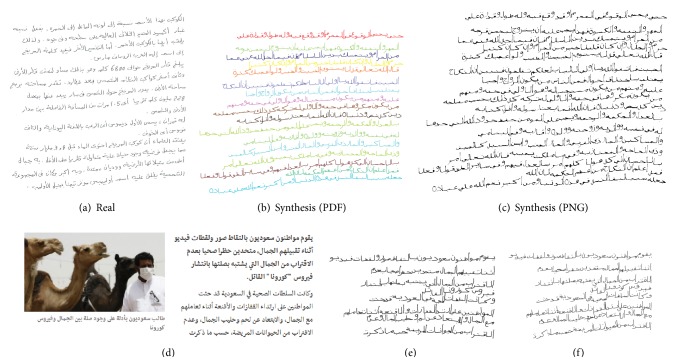
Examples for syntheses of complete text pages: (a) one of the natural handwritings that have been used to compute function *f*; (b) synthesis using optimized *f*_1_ (given by (15)), rendered as PDF; (c) another synthesis using *f*_1_, rendered as png image. More examples inclusive ground truth will be added at http://www.iesk-ardb.ovgu.de/ soon. Text synthesis from a BBC newsletter (d); BBC article about a virus outbreak in Saudi Arabia [5] (e-f); two syntheses of the given paragraph of (d).

Outcomes of the described techniques of text page generation are shown in Figure 14. How to determine a proper baseline function *f*(*x*, *y*_0_) will be discussed in the next section.

### 5.6. Optimization of Baseline Functions

A set of baselines Ξ are *n* sequences of bounding boxes of all PAW within a page, ordered from the first to the last PAW of a line. To validate and optimize synthetic baselines Ξ_*s*_, which are a result of *f*(*x*, *y*_0_), we calculate the correlation *ρ* = corr(Ξ_*r*_, Ξ_*s*_), where Ξ_*r*_ contains 11295 PAW extracted from natural handwritings.

To get the global correlation *ρ*_*g*_, we train a Gaussian Mixture Model (GMM) on Ξ_*r*_. Therefore, features *x* as the average *μ* and sigma *σ* of the normalized space between text lines, *μ*∧*σ* of the angle *ϕ* between a text line and the horizontal, or *μ*∧*σ* of the change of *ϕ* depending on *y*_0_ are used. Subsequently, we use the GMM to calculate the log likelihood log⁡*ℒ*(*θ*∣**x**), where Ξ_*s*_ with **x** belongs to the class of natural baselines *θ*. Now the global correlation can be computed by *ρ*_*g*_ = *n* ^−^1∑^*n*^log⁡*ℒ*(*θ*∣**x**).

To detect lines that have odd curvatures, we also compare each synthetic line with all natural ones. Therefore, we represent each line by *𝔏*, which is a series of the geometrical centers *𝔠* of the PAW-bounding boxes. To ease the comparison, all natural *𝔏*_*r*_ and synthetic *𝔏*_*s*_ lines are normalized and translated, so that their first (rightmost) *𝔠* lies within the origin. For all *m*_Ξ_ centers *𝔠* ∈ *𝔏*_*s*_ we search neighbored *𝔠* ^*a*^, *𝔠* ^*b*^ ∈ *𝔏*_*r*_ that fulfill *𝔠*_*x*_^*a*^ > *𝔠*_*x*_ > *𝔠*_*x*_^*b*^. Now *ρ*_*l*_ = corr(*𝔏*_*s*_, *𝔏*_*r*_) can be calculated:

(14) ρl=log⁡1−∑mΞc−crmΞ,cr=cxa−cxcxb−cxaca+cx−cxbcxb−cxacb.

Using the average ${\bar{\rho }}_{l}$ of the five best matches *ρ*_*l*_ we get $\rho ={\bar{\rho }}_{l}+\rho_{g}$, where *ρ* indicated how proper *f*(*x*, *y*_0_) simulates the shapes of natural baselines. The functions *f*_*i*_ have to be defined manually within the UI; however, an automatically optimization can be initialized subsequently. We defined multiple *f*_*i*_, reflecting different peculiarities that could be observed studying historical and other Arabic handwritings. The function that defines a set of ground truth (nonhistorical) text pages of the IESK-arDB and that was used to generate the syntheses in Figure 14 is *f*_1_(*x*, *y*_0_):

(15) −2q1xy0+y00.7sin⁡2.5x−1.2+q2·sin⁡10x+q3·sin⁡8x+2.

The parameters *q*_*i*_ are redefined by Gaussian distributions *𝒩*_*i*_(*μ*_*i*_, *σ*_*i*_) for each page synthesis. To find the optimal *q*_*i*_, we use genetic programming where *μ*_*i*_, *σ*_*i*_ are the genetic representation and *ρ* is the fitness of an individual. For *f*_1_ we get the parameterization:

(16) q1⟸N0.15,0.84,q2⟸N0.67,0.51,q3⟸N0.35,0.17.

Depending on the defined formula and features of the used Ξ_*r*_, more or less challenging page syntheses can be achieved.

## 6. Results and Discussions

We found that our system is able to synthesize multiple realistic samples for all words that do not include special characters like Hamza over Nabira or ligatures like LamAlif or digits (which can be included when extending the letter database). In the following we propose a method to declare suitable functions for Baseline definitions and validate the applicability of our synthesis outcomes.

### 6.1. Synthesis Evaluation

Since the data synthesis module is built to ease the development and validation of methods that are related to document analysis, it is not only of interest whether syntheses look realistic or not. In fact it is crucial how image processing methods behave when being fed with synthetic instead of natural data. This is investigated in this section, where a method that segments handwritten Arabic words into letters is validated on such data. We chose word segmentation as example, due to its sensitivity to character shapes as well as global features like overlapping PAWs or varying Kashida length.

*Segmentation of Arabic Words.* The segmentation method, which is used for the following experiments, is described in [4]. It is based on topological features and a set of rules that reduces all candidates to a final set of points, which divide two neighbored letters. In contrast to other approaches, candidates are not minima that indicate the middle of a Kashida, but typically the following branch point. 

*Comparison of Real and Synthetic Validation Databases*. The synthetic samples which are created by the proposed approach are meant as training or testing data for different document analysis methods. To investigate whether these syntheses can be used instead or additional to natural samples, we created synthetic samples (png files + ground truth) of all words of the IESK-arDB database [4] that we call IESK-arDB-Syn in the following. We validate the described segmentation method on both databases using cross validation. As shown in Table 4 the detected error rates are comparable. This proves at least that the proposed synthesis method is capable of reflecting those features of natural Arabic words, which are critical for segmentations.

**Figure 15:**
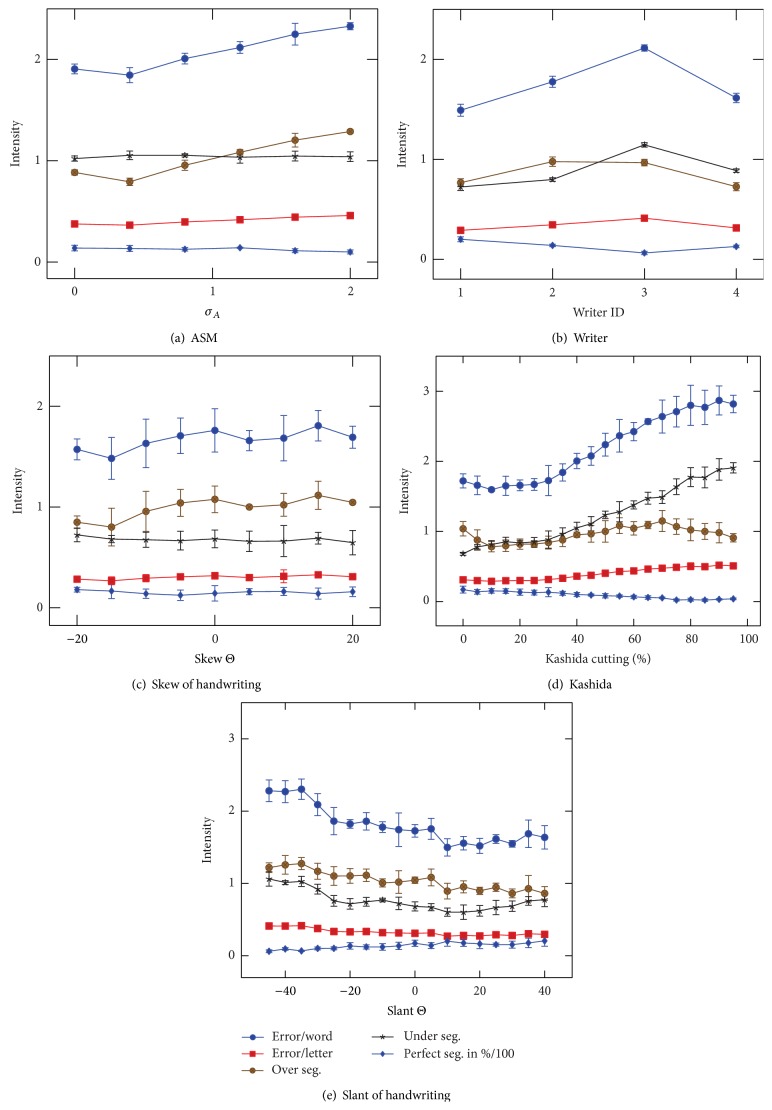
Experiments show the influence of different features on the average error of the word segmentation method.

Furthermore the proposed synthesis approach enables investigating the robustness of the segmentation method against the influence of particular features. Therefore, we built modifications of IESK-arDB-Syn for experiments A and B, using the UI to ensure that only the investigated feature differs for samples of the same writer. The results of these experiments are shown in Figure 15. 

*Experiment A*:* ASM Eigenvector Intensity*. The intensities *c*_*i*_ of the used eigenvectors define the similarity of a computed letter shape **u** and $\bar{x}$, where maximal intensities (*c*_*i*_) often causes unexpected, deformed shapes which are hard to classify. The experiment confirms that eigenvector intensities are also proportional to segmentation error frequency. However, even strongly deformed letter shapes reduce the segmentation results only moderately, since their influence on Kashidas and key features, as branch points, is less than their influence on the signature of the letter curvature, which seems more vital regarding character recognition. 

*Experiment B*:* Writer*. As one can see in Figure 15(b), the segmentation method is quite sensitive to the writer dependent style. Best performance is achieved on writer 1, since letters are written in a proper style and have long Kashidas.

*Experiment C*:* Skew*. Although skew correction is a common preprocessing step, this experiments show that the used segmentation method is robust against a skew of ±20°.

*Experiment D*:* Kashida Length*. Kashidas are the connections of two letters, which have a high variation in case of Arabic handwritings. The experiment shows that a valid segmentation is especially difficult in case of very small Kashidas, since most structures that indicate a potential dividing point are hidden or vanished in such cases. In extreme cases neighbored letters can be written one above the other. This effect could be observed in both natural and synthetic handwritings and makes segmentation very challenging. In contrast to slant and skew variation, Kashida related problems cannot be solved by a simple preprocessing technique. 

*Experiment E*:* Slant*. The used segmentation method does only segment at rows with exactly one foreground pixel. Hence, extreme slants can cause segmentation errors, if the slant causes strongly overlapping ascenders. The experiment shows that a slant of about 20° even slightly improves the segmentation results, which might be caused by the frequent appearance of Alif (ا) in end-form that can be detected more reliable, if it has a positive slant. This effect is weakened when reducing the Kashidas length, though. Finally, the experiment shows that slant correction is not a mandatory but nonetheless useful preprocessing step for the proposed segmentation method, especially for handwritings with strong negative slant.

## 7. Conclusion

We have presented an efficient approach to generate pseudo handwritten Arabic words and text pages, including diacritic marks (dots), from Unicode. Online sample and Active Shape Model based glyphs from multiple writers as well as affine transformations allow to generate various images for a given Unicode string to cover the variability of human handwritings. Data of new writers can be added easily and efficiently, since the definition of manual landmarks is not necessary. Features as the slant can be controlled manually if desired. Interpolation methods and a rendering technique are used to meet the properties of offline handwritings. We investigated the practical applicability of the synthesis by validating a segmentation algorithm on natural and synthetic data getting comparable results.

In our future work we are going to extend the used alphabet, acquire letter samples from more writers, and reduce the amount of synthesized words by clustering techniques as affinity propagation. This will help to synthesize compact but representative databases and use them to train and test methods for handwriting recognition and document analysis approaches.

## Figures and Tables

**Algorithm 1:**
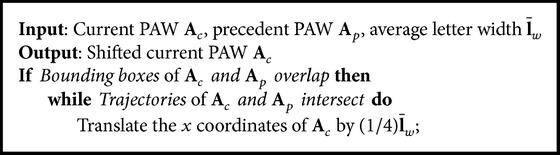
Solving intersections of neighbored PAWs.

**Table 1:** Arabic alphabet, Naskh style.

	i	e	m	b
Alif	ا	ـا		
Ba	ب	ـب	ـبـ	بـ
Ta	ت	ـت	ـتـ	تـ
Tha	ث	ـث	ـثـ	ثـ
Jim	ج	ـج	ـجـ	جـ
Ha	ح	ـح	ـحـ	حـ
Kha	خ	ـخ	ـخـ	خـ
Dal	د	ـد		
Thal	ذ	ـذ		
Ra	ر	ـر		
Zai	ز	ـز		
Sin	س	ـس	ـسـ	سـ
Shin	ش	ـش	ـشـ	شـ
Sad	ص	ـص	ـصـ	صـ
Dhad	ض	ـض	ـضـ	ضـ
Taa	ط	ـط	ـطـ	طـ
Dha	ظ	ـظ	ـظـ	ظـ
Ayn	ع	ـع	ـعـ	عـ
Ghayn	غ	ـغ	ـغـ	غـ
Fa	ف	ـف	ـفـ	فـ
Qaf	ق	ـق	ـقـ	قـ
Kaf	ك	ـك	ـكـ	كـ
Lam	ل	ـل	ـلـ	لـ
Mim	م	ـم	ـمـ	مـ
Nun	ن	ـن	ـنـ	نـ
He	ه	ـه	ـهـ	هـ
Waw	و	ـو		
Ya	ي	ـي	ـيـ	يـ

**Table 2:** Parameters and tresholds.

Parameter	Explanation	Value
*r*	Number of samples used to compute an ASM	50
*m*	Number of points of letter sample trajectory $\overset{´}{x}$ before approximation	$\overset{-}{m}=266.8$
*n*	Number of points of a approximated sample **x**	25
*ς*	Controlling the space between words	user defined
*α*_*r*_	Word rotation	±45°
*α*_*s*_	Word slant	±45°
*ϖ*	Controlling the sharpness of synthesis contours	0–10
*ω*_*P*_	Width of synthesized lines in points	1–10

**Table 3:** Syntheses using ASMs and sample based glyphs of writer 1.

$\sigma_{A}\sim \bar{\left|c_{j}\right|}\sqrt{\lambda_{i}}$	Result
0	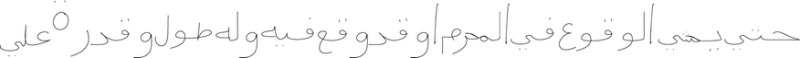
1	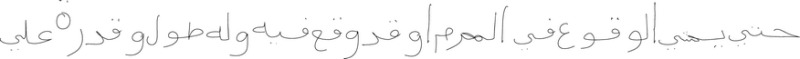
2	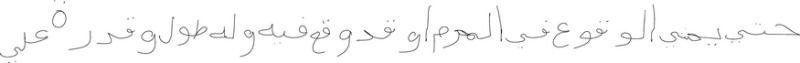
3	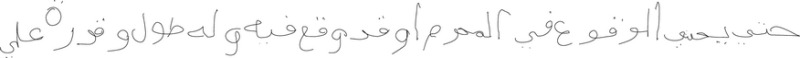
Samples	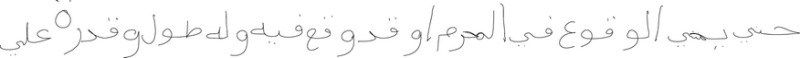

**Table 4:** Validation of the segmentation method.

Database	IESK-arDB		IESK-arDB-Syn	
Number of word samples	2540		9000	
Measure	*μ*	*σ*	*μ*	*σ*
Error per word	1.67	0.13	1.74	0.024
Error per letter	0.35	0.026	0.34	0.0045
Over segmentation (per word)	0.79	0.097	0.86	0.0066
Under segmentation (per word)	0.87	0.071	0.88	0.019
Perfect segmentation (per word)	0.17	0.0019	0.13	0.0067
